# Supplementary material for: Measuring language lateralisation with different language tasks: a systematic review
Source: PeerJ. 2017 Oct 24;5:e3929. doi: 10.7717/peerj.3929 (PMC5659218; doi:10.7717/peerj.3929)
Supplement: Supplemental Information 1 [file peerj-05-3929-s001.doc]

| **Section/topic** | **#** | **Checklist item** | **Reported on page #** |
| --- | --- | --- | --- |
| **TITLE** | | |  |
| Title | 1 | Identify the report as a systematic review, meta-analysis, or both.  Title: Measuring language lateralisation with different language tasks: A **systematic review** | 1 |
| **ABSTRACT** | | |  |
| Structured summary | 2 | Provide a structured summary including, as applicable: background; objectives; data sources; study eligibility criteria, participants, and interventions; study appraisal and synthesis methods; results; limitations; conclusions and implications of key findings; systematic review registration number.  Note that this review is registered as a protocol on open science framework, no registration number provided. | 2 |
| **INTRODUCTION** | | |  |
| Rationale | 3 | Describe the rationale for the review in the context of what is already known.  *Multiple language tasks have been used with fMRI. At first glance, the literature appears to support the notion that language laterality is not unitary, because we can see differences between tasks in the strength of the laterality measurements they yield. However, the reasons for such variability in LI strength across language tasks can be debated; could it simply be an artefact of more trivial differences in task design, or does it reveal something fundamental about the hemispheric organisation of different components of language? Answering such questions requires optimisation of protocols for LI measurement, to ensure that variability in LIs measured for different language functions cannot be attributed to differences in task sensitivity or measurement error.* | 4 |
| Objectives | 4 | Provide an explicit statement of questions being addressed with reference to participants, interventions, comparisons, outcomes, and study design (PICOS).  *This systematic review aims to assess evidence on the robustness of laterality measured using fMRI with different language tasks, from studies published between 2000 and 2016. This is done with a view to providing some guidance on optimizing variables such as region of interest and baseline task on a task-by-task basis. Such optimization will be important before tasks can be used to systematically probe patterns of co-lateralisation and independent lateralisation of different language functions.* | 5 |
| **METHODS** | | |  |
| Protocol and registration | 5 | Indicate if a review protocol exists, if and where it can be accessed (e.g., Web address), and, if available, provide registration information including registration number.  *A protocol for this systematic review has been registered on Open Science Framework and can be found at https://osf.io/5vmpt/.* | 5 |
| Eligibility criteria | 6 | Specify study characteristics (e.g., PICOS, length of follow-up) and report characteristics (e.g., years considered, language, publication status) used as criteria for eligibility, giving rationale.  *We selected papers using fMRI to study language lateralisation published between 2000 and 2016, using the following criteria: (1) the paper reported LIs for language calculated using fMRI; (2) the paper studied healthy monolingual adults; and (3) if both patients and healthy controls were studied, the data for controls were reported separately. Papers were excluded if: (1) they exclusively studied structural asymmetries, children or bilingualism; or (2) they used language tasks with non-European languages. The search was restricted to studies of healthy, monolingual, adult participants to reduce heterogeneity within our study sample.* | 5 |
| Information sources | 7 | Describe all information sources (e.g., databases with dates of coverage, contact with study authors to identify additional studies) in the search and date last searched.  *The following search terms were used to search papers published between 2000 and 2016 in Web of Science… This was last searched on 05/12/16* | 6 |
| Search | 8 | Present full electronic search strategy for at least one database, including any limits used, such that it could be repeated.  *The following search terms were used to search papers published between 2000 and 2016 in Web of Science: laterali* OR asymmetr* OR dominance; AND language OR reading; AND fMRI OR functional MRI OR functional magnetic resonance imaging OR functional MR OR function MRI; NOT schizophrenia; NOT development*; NOT child*; NOT bilingual*. This was last searched on 05/12/16.* | 6 |
| Study selection | 9 | State the process for selecting studies (i.e., screening, eligibility, included in systematic review, and, if applicable, included in the meta-analysis).  *Titles and abstracts of the resulting 90 papers were then screened by two of the review authors (Abigail Bradshaw and Zoe Woodhead), followed by full-text scans to determine whether the inclusion criteria were met. Selected lists were compared between reviewers and any discrepancies discussed and a mutual decision made. This resulted in the selection of 34 papers. To further search the literature, we screened papers citing these 34 articles. A further 50 articles were identified as meeting our criteria, yielding a total of 84 papers. A final check of papers led to the discounting of 7 papers deemed to not sufficiently meet criteria, with a further paper being discounted during conductance of the review.*  Criteria for selecting suitable papers with LI data to be plotted in forest plots:  *To avoid the potential confound of heterogeneity in samples in terms of handedness, we included mean LIs reported either from only right handed participants, or from mixed handedness samples where the relative proportion of left and right handers was representative of the general population (around 10% left handed, 90% right handed). We excluded LIs reported from studies that selected a participant group on the basis of their pre-known lateralisation. Where more than one frontal LI was reported from a study, IFG LIs were selected; where more than one temporoparietal LI was reported, that LI calculated from the largest area of temporoparietal cortex was selected.* | 6 (for systematic review), 7 (for forest plot data) |
| Data collection process | 10 | Describe method of data extraction from reports (e.g., piloted forms, independently, in duplicate) and any processes for obtaining and confirming data from investigators.  *Information on variables of interest for each study were collected and managed using REDCap electronic data capture tools (Harris et al., 2009) hosted at Oxford University. REDCap (Research Electronic Data Capture) is a secure, web-based application designed to support data capture for research studies, providing: 1) an intuitive interface for validated data entry; 2) audit trails for tracking data manipulation and export procedures; 3) automated export procedures for seamless data downloads to common statistical packages; and 4) procedures for importing data from external sources. The full database can be found in Appendix 2. A summary table drawn from this database with the key outcomes of interest for this paper is provided in Appendix 3.* | 6 |
| Data items | 11 | List and define all variables for which data were sought (e.g., PICOS, funding sources) and any assumptions and simplifications made.  *For each paper, we recorded: sample size and handedness, the type of fMRI design used, the activity measures used for LI calculation, the threshold level chosen, the use of global or regional LI calculation, the specific regions considered, the language and baseline tasks used, the use of a single or a combined task analysis and the task difficulty.* | 6-7 |
| Risk of bias in individual studies | 12 | Describe methods used for assessing risk of bias of individual studies (including specification of whether this was done at the study or outcome level), and how this information is to be used in any data synthesis.  We did not assess risk of bias, as this review is not concerned with clinical trials and so issues of randomisation and blinding were not relevant. | N.A. |
| Summary measures | 13 | State the principal summary measures (e.g., risk ratio, difference in means).  *The variable nature of the methods and measures reported by different papers did not permit performance of a meta-analysis. Instead, to illustrate the strength of laterality measured across different language tasks, we produced forest plots showing the mean and 95% confidence intervals of LI values reported in the studies, as well as their associated methods of LI calculation, region(s) of interest, and language and baseline tasks (Figures 2, 3 and 4).* | 7 |
| Synthesis of results | 14 | Describe the methods of handling data and combining results of studies, if done, including measures of consistency (e.g., I^2^) for each meta-analysis. | N.A. |

Page 1 of 2

| **Section/topic** | **#** | **Checklist item** | **Reported on page #** |
| --- | --- | --- | --- |
| Risk of bias across studies | 15 | Specify any assessment of risk of bias that may affect the cumulative evidence (e.g., publication bias, selective reporting within studies). | N.A. |
| Additional analyses | 16 | Describe methods of additional analyses (e.g., sensitivity or subgroup analyses, meta-regression), if done, indicating which were pre-specified. | N.A. |
| **RESULTS** | | |  |
| Study selection | 17 | Give numbers of studies screened, assessed for eligibility, and included in the review, with reasons for exclusions at each stage, ideally with a flow diagram.  See checklist item 9. PRISMA flow diagram included as a figure. | 6 and Figure 1 (PRISMA flow diagram) |
| Study characteristics | 18 | For each study, present characteristics for which data were extracted (e.g., study size, PICOS, follow-up period) and provide the citations.  Data extracted from each study can be found in Appendix S2, an excel spread-sheet drawn from the online database on Redcap software used to record information for each study. A summary table drawn from this database of main outcomes of interest for this paper can be found in Table S3. | See supplementary tables S2 and S3. |
| Risk of bias within studies | 19 | Present data on risk of bias of each study and, if available, any outcome level assessment (see item 12). | N.A. |
| Results of individual studies | 20 | For all outcomes considered (benefits or harms), present, for each study: (a) simple summary data for each intervention group (b) effect estimates and confidence intervals, ideally with a forest plot. | See figures 2, 3 and 4. |
| Synthesis of results | 21 | Present results of each meta-analysis done, including confidence intervals and measures of consistency. | N.A. |
| Risk of bias across studies | 22 | Present results of any assessment of risk of bias across studies (see Item 15). | N.A. |
| Additional analysis | 23 | Give results of additional analyses, if done (e.g., sensitivity or subgroup analyses, meta-regression [see Item 16]). | N.A. |
| **DISCUSSION** | | |  |
| Summary of evidence | 24 | Summarize the main findings including the strength of evidence for each main outcome; consider their relevance to key groups (e.g., healthcare providers, users, and policy makers). | 24-25 |
| Limitations | 25 | Discuss limitations at study and outcome level (e.g., risk of bias), and at review-level (e.g., incomplete retrieval of identified research, reporting bias). | N.A. |
| Conclusions | 26 | Provide a general interpretation of the results in the context of other evidence, and implications for future research.  *This review has highlighted the high level of variation and inconsistency in the strength and reliability of laterality measured using different language tasks, which presents a challenge when designing laterality experiments. The current state of the literature is such that it is difficult to draw clear conclusions that can be used to guide task selection. This review highlights the need for more research that systematically compares laterality across different tasks in within-subject designs, with rigorous matching of non-linguistic aspects of task design.* | 24 |
| **FUNDING** | | |  |
| Funding | 27 | Describe sources of funding for the systematic review and other support (e.g., supply of data); role of funders for the systematic review.  *This work was supported by an Advanced Grant awarded by the European Research Council (project 694189 - Cerebral Asymmetry: New directions in Correlates and Etiology – CANDICE). Dorothy Bishop is funded by programme grant 082498/Z/07/Z from the Wellcome Trust.* | 26 |

*From:*  Moher D, Liberati A, Tetzlaff J, Altman DG, The PRISMA Group (2009). Preferred Reporting Items for Systematic Reviews and Meta-Analyses: The PRISMA Statement. PLoS Med 6(7): e1000097. doi:10.1371/journal.pmed1000097

For more information, visit: **www.prisma-statement.org**.

Page 2 of 2
