# Supplementary material for: Measuring language lateralisation with different language tasks: a systematic review
Source: PeerJ. 2017 Oct 24;5:e3929. doi: 10.7717/peerj.3929 (PMC5659218; doi:10.7717/peerj.3929)
Supplement: Supplemental Information 5 — Summary of key outcomes of interest recorded for each study drawn from the full database S2. [file peerj-05-3929-s005.pdf]

Lateralisation

Data Exports, Reports, and Stats

VIDEO: How to use Data Exports, Reports, and Stats

Summary table Language tasks

**Article authors** (article_authors)	**Publication year** (year_of_publication)	**Sample size** (sample_size)	**Sample_Handedness** (sample_handedness)	**List specific language tasks used** (specific_language_tasks)	**Specific baseline tasks used** (baseline_tasks)	**Key conclusions** (study_conclusions)
Abbott, Waites, Lillywhite and Jackson	2010	34	Unknown (6)	Letter cued word generation	Visual cross fixation (rest)	Laterality should not be determined at a single threshold but using thresholds that yield a fixed number of active voxels i.e. an individually determined variable threshold, as this is more robust. Argue for the utility of an approach to dominance classification in which an LI distribution across voxel counts for a subject can be compared to a normative distribution.
Adcock, Wise, Oxbury, Oxbury, Matthews	2003	12	Only Right handers (1)	Visually letter cued covert word generation	Cross fixation	fMRI LIs are highly reproducible. Magnitude LIs were more robust than extent LIs in terms of lower variability across testing sessions. ROI had little effect on strength of LI.
Baciu, Juphard, Cousin, Le Bas	2005	10	Only Right handers (1)	Word generation task,<br><br>Rhyme detection task	Passive cross fixation for word generation.<br><br>Visual detection task for rhyme detection- perceptual judgement on unreadable words.	The rhyme detection task appears more robust than the word generation task in that LI values were more resistant to the effects of normalisation, smoothing and clustering. For rhyme detection, FM appeared superior to LIM in terms of correlation with manual LIs; neither method correlated with manual LIs for word generation.
Bethmann, Tempelmann, Bleser, Scheich, Brechmann	2007	30	Both right, left and ambidextrous (5)	Semantic decision task (from Fernandez et al, 2001)	Perceptual matching decision- decide if two non-word letter strings were identical.	Classifying language dominance based on a single ROI is not always appropriate for characterising an individual's pattern of language laterality, in light of cases of crossed regional dominance. LI extent and magnitude measures did not differ significantly in magnitude and were strongly correlated.
Berl, Zimmaro, Khan, Dustin, Ritzl, Duke et al.	2014	118	Only Right handers (1)	Semantic definition verification	Listen to reverse speech and identify tones.	A data-driven clustering method indicated the existence of meaningful categories of language dominance versus the arbitrary categorisation cut-offs traditionally used. Laterality should be assessed on a regional rather than a global level.
Fernandez, de Greiff, von Oertzen, Reuber, Lun, Klaver et al.	2001	12	Only Right handers (1)	Semantic decision task	Perceptual matching decision- decide if two non-word letter strings were identical.	Laterality analyses based on offline and online image processing were highly correlated. For this semantic decision task, temporoparietal mean LI was higher than frontal mean LI.
Jansen, Menke, Sommer, Forster, Bruchmann, Hempleman, Weber, Knecht.	2006	10	Both right and left handers (4)	Word generation,<br><br>Semantic decision,<br><br>Picture naming.	Word generation- silent repetition of non-word 'baba'.<br><br>Semantic decision- perceptual matching for non-word letter strings.<br><br>Picture naming- cross fixation.	LI extent and LI magnitude measures have similar reproducibility.<br>But, recommended that the most reproducible and robust LIs could be obtained using magnitude of signal change from those voxels exceeding a pre-set threshold value within a defined ROI.<br>The word generation task may be just as reproducible as CTA, which are both more reproducible than semantic decision.
Binder, Swanson, Hammeke, Sabsevitz	2008	26	Only Right handers (1)	Passive word listening,<br><br>Semantic decision,<br><br>Phoneme decision.	Rest.<br><br>Passive tone listening.<br><br>Active tone decision- decide if sequence contained two 750Hz tones.	Resting baselines should not be used for speech comprehension protocols, due to resting activation of conceptual representations. Overall, a semantic decision- tone decision protocol was optimal in terms of producing the strongest and most consistent lateralisation.
Allendorfa, Hernando, Hossain, Nenert, Holland, Szaflarski	2016	214	Both right, left and ambidextrous (5)	Covert verb generation.	Bilateral finger tapping in response to a 400 Hz tone.	Laterality strength did not differ between right and left handers for verb generation; was on average 0.44 for right handers and 0.35 for left handers.
Backes, Deblaere, Vonck, Kessels, Boon, Hofman, Wilmink et al.	2005	9	Only Right handers (1)	Word generation,<br><br>Text reading.	Word generation- passive cross fixation.<br><br>Text reading- reading of text composed of pronounceable non-words.	Strong mean LIs obtained for both word generation and text reading covert paradigms.
Brennan, Whalen, Branco, O'Shea, Norton, Golby	2007	7	Only Right handers (1)	Object naming,<br><br>Number counting	Passive fixation,<br><br>Viewing of non-semantic nonsense objects (perceptual control)	Automated language paradigms appear to be more weakly lateralising than non-automated speech tasks.
Clements, Rimrodt, Abel, Blankner, Mostofsky, Pekar et al.	2006	30	Only Right handers (1)	Rhyming decision	Perceptual decision- decide if two sets of oriented lines had the same pattern or not.	Strong laterality obtained for a rhyming decision task using an active perceptual decision baseline within a frontal ROI.
Cousin, Peyrun, Pichat, Lamalle, Le Bas, Baciu	2007	11	Only Right handers (1)	Rhyme detection task,<br><br>Semantic Categorization task.	Visual detection task- perceptual judgement of whether words of unreadable characters had at least one character which overshot the others.	The flip method can be used to assess the significance of hemispheric asymmetries across different areas, and whether such asymmetries relate to the condition of interest or to the control task. Taking the control task into account when comparing the hemispheres yielded more left lateralised areas.
Deblaere, Backes, Hofman, Vandemaele, Boon, Vonck et al.	2002	9	Only Right handers (1)	Word generation,<br><br>Semantic decision,<br><br>Reading,<br><br>Naming.	Cross fixation for word generation.<br><br>Vowel decision (decide if vowel /a/ is in vowel string) for semantic decision.<br><br>Reading of nonsense word text for reading.<br><br>Line task (name position of intersection of four lines) for naming.	Rank ordering of language tasks from strongest LI to weakest: Word generation, semantic decision, reading, naming. Confirms the strongly lateralising power of word generation, and provides some suggestions of the effects of baseline on LI; in particular, argued their baselines for the other tasks may have engaged language processes to too high an extent.
Dodoo-Schittko, Rosengarth, Doenitz, Greenlee	2012	11	Only Right handers (1)	Verb generation,<br><br>Antonym generation,<br><br>Verb congruency decision,<br><br>Antonym decision.	Generation control: Required to subvocally change the order of two syllables of a pseudoword.<br><br>Decision control: Colour rule task which required discovery of a rule which associated colours with consonants within letter rows and decision as to whether this rule is conformed to or violated by the current set of letter rows.	Demonstrated strong effects on laterality of both single versus combined task analysis, and resting baselines versus active control conditions. An active control task may make a STA more strongly lateralised than a CTA using resting baselines. In general, CTA yields the strongest LIs. Generation tasks yield stronger LIs than decision tasks, but the latter may be more robust against threshold dependence.
Doucet, Pustina, Skidmore, Sharan, Sperling,Tracy	2015	23	Both right and left handers (4)	Verb generation	Passive fixation.	Demonstration of strong laterality (0.59) for word generation task.
Drager, Jansen, Bruchmann, Forster, Pleger, P'zwitserlood et al.	2004	14	Only Right handers (1)	Word stem completion	Silent repetition of a visually presented word.	Overall, task difficulty had no significant effect on lateralisation within a frontal ROI for a verbal fluency task.

Article authors (article_authors)	Publication year (year_of_publication)	Sample size (sample_size)	Sample_Handedness (sample_handedness)	List specific language tasks used (specific_language_tasks)	Specific baseline tasks used (baseline_tasks)	Key conclusions (study_conclusions)
Fesl, Bruhns, Rau, Wiesmann, Ilmberger, Kegel et al.	2010	39	Both right, left and ambidextrous (5)	Reversed association task.	Phoneme sequence listening/detection- listen to unintelligible phoneme sequences and respond afterwards by producing the word 'nothing' (matches number of overt responses required in language task).	This novel free reversed association task produced strong and reliable lateralisation within multiple ROIs. The most reliable LIs were obtained using a global ROI and variable (as opposed to fixed) thresholds.
Gaillard, Balsamor, Xu, Grandin, Braniecki, Papero et al.	2002	22	Only Right handers (1)	Read response naming	Passive viewing of dot patterns displayed one at a time- matched the task sentences for sentence length.	The read response naming task is capable of yielding strong laterality in both frontal and temporal ROIs. This task is thus useful for lateralising temporal receptive language areas involved in reading comprehension.
Gaillard, Sachs, Whitnah, Ahmad, Balsamo, Petrella et al.	2003	22	Only Right handers (1)	Semantic fluency.	Silent rest.	Demonstration that semantic fluency can yield strong lateralisation particularly in frontal but also in temporal ROIs.
Haberling, Badzakova-Trajkov, Corballis	2011	60	Both right and left handers (4)	Word generation	Cross fixation	Demonstration of strong laterality for word generation within a frontal ROI.
Haberling, Steinemann, Corballis	2016	94	Both right and left handers (4)	Word generation,<br><br>Synonym decision	Word generation: Passive cross fixation.<br><br>Synonym decision- perceptual decision task (decide if two letter strings were identical).	Laterality was almost identical across ROIs and tasks, indicating that lateralization is no more pronounced for production than for comprehension. However, cases of crossed laterality were found.
Harrington, Buonocore, Farias.	2006	10	Only Right handers (1)	Verb generation,<br><br>Semantic decision,<br><br>Confrontation naming,<br><br>Story listening,<br><br>Visual and oral sentence comprehension.	Verb generation, story listening, oral sentence comprehension- passive listening to backwards speech.<br><br>SD- perceptual decision on letters (upper or lower case).<br><br>Naming- attend to visual patterns of lines.<br><br>Visual sentence comprehension- attend to strings of lines (similar length to sentences).	Overall, the reproducibility of LIs within a given ROI depends on the task used; reproducible LIs can be found within temporal as well as frontal ROIs, provided the right task is used (e.g. story listening). Magnitude LIs were more reproducible than extent LIs. Overall, verb generation was superior in terms of strength and reproducibility of LIs across ROIs (comparable to reproducibility of CTA).
Hernandez, Andersson, Edjlali, Hommet, Cottier, Destieux et al.	2013	16	Only Right handers (1)	Rhyming task.	Font matching task- decide if pairs of unreadable fonts were matching or non-matching.	Rhyming decision task yields significant lateralisation across multiple ROIs using the flip method of interhemispheric comparison.
Hund-Georgiadis, Lex, von Cramon.	2001	14	Only Right handers (1)	Semantic encoding/decision,<br><br>Lexical encoding/decision.	Fixation,<br><br>Visual perceptual decision- decide if upper or lower case,<br><br>Auditory perceptual decision- decide if speaker is male or female.	Highest laterality was obtained with semantic decision compared to perceptual decision. Active baseline yielded higher LIs than passive fixation baseline. Weak laterality (i.e. bilateral activation) seen for lexical encoding task, which may be attributable to engagement of syntactic processes. No significant effect of stimulus modality, task performance, nor response hand found.
Hund-Georgiadis, Lex, Friederici, von Cramon.	2002	34	Both right and left handers (4)	Semantic encoding/decision,<br><br>Lexical encoding/decision.	Rest,<br><br>Perceptual encoding task- decide if word is in upper or lower case letters.	Strong laterality found for a semantic decision task vs perceptual encoding baseline across both frontal, temporal and global LIs (i.e. no effect of region). Weaker laterality found for a lexical encoding task, and for both tasks when a fixation baseline was used.
Hunter, Brysbaert.	2008	10	Only Left handers (2)	Word generation.	Silent repetition of non-word (dada)	Strong laterality was yielded by the word generation task within Broca's area within a sample of left handers; weak laterality (bilaterality) was only found in 2 out of 10 subjects, with the rest showing clear and strong asymmetry.
Jensen-Kondering, Ghobadi, Wolff, Jansen, Ulmer.	2012	20	Only Right handers (1)	Word generation,<br><br>Verb generation,<br><br>Semantic fluency,<br><br>Semantic violation/decision,<br><br>Lexical decision.	Visual baseline- Perceptual decision on direction of arrow within line of dashes.<br><br>Acoustic baseline- Tone decision on whether sequence of two tones was ascending or descending in frequency.	The strongest lateralisation within a temporal ROI was found with an auditorily presented word generation task. Strong lateralisation within temporal areas also found using visual semantic fluency and lexical decision. Overall, effect of stimulus modality and ROI was task dependent. However, across tasks and ROIs, LIs based on voxel counts were always higher than LIs based on voxel signal magnitude (t-value).
Kennan, Kim, Maki, Koizu mi, Constable.	2002	6	Both right and left handers (4)	Syntactic/semantic violation detection	Line decision task.	This sentence comprehension task requiring the detection of syntactic and semantic errors yielded relatively strong lateralisation when a global LI was used.
Kleinhans, Mueller, Cohen, Courchesne.	2008	14	Both right and left handers (4)	Letter fluency,<br><br>Semantic fluency.	Repetition of the word 'nothing'.	Found significantly stronger lateralisation for a letter fluency task compared to a semantic fluency task using a frontal ROI; attributed to greater recruitment of right prefrontal cortex during semantic fluency.
Knecht, Jansen, Frank, van Randenborgh, Sommer, Kanowski et al.	2003	14	Both right and left handers (4)	Word generation	Silent nonword repetition ('baba')	For word generation, no differences were found in the extent of variability in LIs between a left dominant and a right dominant group (classified by fTCD). No evidence that one group showed greater bilaterality than the other. Demonstration that some individuals do show right dominance for word generation, in a way concordant with fTCD findings.
Krinik, Lehericy, Duffau, Capelle, Chainay, Cornu, et al.	2003	6	Only Right handers (1)	Semantic fluency.	Rest.	LIs based on voxel counts within the SMA are generally weaker and more variable between individuals than LIs based on whole hemispheres, using a verbal fluency task.
Lohmann, Deppe, Jansen, Schwindt, Knecht.	2004	1	Only Right handers (1)	Word generation.	Rest.	Task repetition led to 'pseudoincreases' in bilaterality; this was markedly demonstrated in LIs based on voxel counts. LIs based on signal magnitude showed no consistent trend over repeated testing, but were generally highly variable. Note: Based on a single subject.
Mazoyer, Zago, Jobard, Crivello, Joliot, Perchey et al.	2014	297	Both right and left handers (4)	Sentence generation.	Covertly recite the months of the year when a scrambled image was presented instead of a cartoon. These trials were randomly interspersed in an event-related paradigm.	A gaussian modelling approach can be used to identify dominance groups without the need for arbitrary cut-offs. 'Atypical' language laterality can be split into 'ambilateral' and 'strongly atypical' groups. Strong right hemisphere dominance was only found in left handers; otherwise, no relationship between handedness and laterality.
Miro, Ripolles, Lopez-Barroso, Vila-Ballo, Juncadella, de Diego-Balaguer et al.	2014	19	Only Right handers (1)	Passive sentence listening.<br><br>Passive pseudosentence listening.	Rest.	This passive sentence listening task was poor at producing lateralised activity within temporal areas when compared to rest.
Morrison, Churchill, Cuimano, Schweizer, Das, Graham.	2016	12	Both right and left handers (4)	Word generation,<br><br>Rhyming decision.	Word gen- wrote varying lengths of symbol strings (e.g. 888, 8888, 88).<br><br>Rhyming- perceptual decision on whether pairs of lines were matching in orientation or not.	Reliability of fMRI LI was task dependent. Rhyming decision laterality was more reproducible than that produced by word generation, in terms of concordance of language dominance and correlation between LI values across two testing runs.
	2015	25	Only Right handers (1)	Word generation	Rest.	

Article authors (article_authors)	Publication year (year_of_ publication)	Sample size (sample_ size)	Sample_Handedness (sample_handedness)	List specific language tasks used (specific_language_ tasks)	Specific baseline tasks used (baseline_tasks)	Key conclusions (study_conclusions)
Nadkarni, Andreoli, Nair, Yin, Young, Kundu et al.						Threshold level did not affect dominance categorisation but resulted in increasing LI values with increasing threshold levels.
Niskanen, Kononen, Villberg, Nissi, Ranta-aho, Saisanen, et al.	2012	20	Both right, left and ambidextrous (5)	Word generation (WG),<br><br>Word pair category task (WP),<br><br>Phoneme decision task (PD),<br><br>Responsive naming (RN),<br><br>Sentence comprehension (SC).	WG- fixation and finger tapping task.<br>WP- tone decision task (which of a pair was higher).<br>PD- perceptual decision task (line drawings).<br>RN- view varying rows of hashes (#).<br>SC- tone decision task (did sequence contain 2 high pitch tones).	Overall, concluded that the optimal protocol for measuring language lateralisation with fMRI used a combined task analysis including word generation, read response naming and sentence comprehension. This combination includes both visual and auditory tasks and produced strong and consistent lateralisation.
Ocklenburg, Hugdahl, Westerhausen	2013	29	Only Right handers (1)	Word generation,<br><br>Passive speech listening.	Rest (subjects told to focus on the sound of the fMRI scanner).	Strength of laterality depended largely on task and region. Strongest laterality found for word generation within frontal ROI. Reduced LI values were seen for passive speech listening compared to word generation and for temporal compared to frontal ROIs. Particularly high level of rightward asymmetry seen for passive speech listening within the temporal lobe. Correlation between the two tasks was seen for frontal but not for temporal LIs.
Orellana, Visch-Brink, Vernooji, Kalloe, Satoer, Vincent, et al.	2015	20	Both right and left handers (4)	Verb generation task.	Passive listening to high and low tones.	Demonstrated crossed lateralisation for cerebral versus cerebellar LIs, irrespective of whether subjects were typically or atypically lateralised for language.
Partovi, Jacobi, Rapps, Zipp, Karimi, Rengier et al.	2012b	14	Only Right handers (1)	Semantic fluency,<br><br>Sentence generation.	Not specified- assume rest used as baseline	Strong laterality found for word generation and sentence generation tasks within frontal and temporal ROIs. Stronger laterality found in frontal than temporal ROI for word generation, but almost identical for sentence generation across ROIs.
Partovi, Konrad, Karimi, Rengier, Lyo, Zipp et al.	2012a	14	Only Right handers (1)	Covert semantic fluency,<br><br>Overt semantic fluency,<br><br>Covert sentence generation,<br><br>Overt sentence generation.	Cross fixation.	Covert paradigms yield stronger laterality than overt paradigms. Covert sentence and word generation tasks yield strong and highly reproducible LIs. Covert sentence generation yielded identical mean LI values within frontal and temporal ROIs, whereas covert word generation yielded stronger laterality in frontal than temporal ROIs.
Perlaki, Horvath, Orsi, Aradi, Auer, Varga et al.	2013	16	Only Left handers (2)	Word generation	Rest.	Demonstration of both strong left and right lateralisation in a sample of left handers using the word generation task and a frontal ROI. 5/16 subjects were right dominant, 10/16 left dominant, 1/16 bilateral.
Pravata, Sestieri, Mantini, Briganti, Colicchio, Marra et al.	2011	12	Only Right handers (1)	Verb generation.	Fixation/rest.	Stronger lateralisation found for verb generation task when using a regional rather than a global LI.
Propper, O'Donnell, Whalen, Tie, Norton, Suarez et al.	2010	25	Both right and left handers (4)	Antonym generation	Fixation/rest.	Demonstrated moderate laterality for an antonym generation task using a histogram method of LI calculation. Greater laterality seen for frontal (0.47) than for temporal (0.26) ROI. LI depended on handedness (but inconsistent pattern across ROIs).
Ramsey, Sommer, Rutten, Kahn.	2001	16	Both right and left handers (4)	Verb generation,<br><br>Antonym generation,<br><br>Semantic decision,<br><br>Reversed spelling semantic decision.	Generation tasks- passive cross fixation.<br>Decision tasks- button presses cued by dots on screen.	LI was task and threshold dependent, but not ROI dependent. Overall, CTA provided the best lateralisation in terms of strength, variability/consistency and robustness against changes in threshold level. Generation tasks yielded stronger laterality than decision tasks. VG LIs were reproducible at a high but not a low threshold.
Razafimandimby, Maiza, Herve, Lecardeur, Delamillieure, Brazo et al.	2007	10	Only Right handers (1)	Story listening.	Passive listening to a factual story in a non-native unknown language (Tamil).	Reported reproducible leftward lateralisation for passive story listening within a combined frontal and temporal ROI.
Ruff, Brennan, Peck, Hou, Tabar, Brennan, et al.	2008	7	Only Right handers (1)	Verb generation,<br><br>Phonemic fluency,<br><br>Semantic fluency.	Cross fixation.	A verb generation task was found to be optimal for lateralisation in terms of strength and consistency of lateralization across threshold levels. Greater variability across thresholds and weaker laterality was found for semantic fluency (in some cases, dominance classification was threshold dependent).
Rutten, Ramsey, van Rijen, van Veelen	2002	9	Both right and left handers (4)	Verb generation,<br><br>Antonym generation,<br><br>Picture naming.	Cross/blank screen fixation.	Reproducibility of LIs depended on task and region, but was relatively independent of threshold. CTA produced the most robust and reliable lateralisation. Significantly stronger laterality was found for regional versus global LIs. Verb generation was reliable, whereas picture naming and antonym generation were not. However, strength of laterality did not differ significantly between tasks.
Sanjuan, Bustamante, Forn, Ventura-Campos, Barros-Loscertales, Martinez et al	2010	18	Only Right handers (1)	Verb generation,<br><br>Word generation.	VG- silent repetition of letters.<br><br>WG- silent repetition of word 'casa' (Spanish for house).	Stronger lateralisation was found for a verb generation task over a word generation task within a frontal ROI; however LIs for the tasks were significantly correlated.
Sanjuan, Forn, Ventura-Campos, Rodriguez-Pujadas, Garcia-Porcar, Belloch et al.	2010	22	Only Right handers (1)	Sentence verification task.	Phoneme decision task- decide if there is a letter 'A' within a pair of letters presented auditorily, by raising their right (true) or left (false) hand.	A sentence verification task using an active phoneme decision baseline yielded strong laterality in temporoparietal areas (weaker in frontal areas). All subjects were left dominant within temporoparietal ROI (right handed sample). This is thus a suitable task for assessing lateralisation of receptive language function.
Seghier, Lazeyras, Pegna, Annoni, Zimine, Mayer, et al.	2004	26	Only Right handers (1)	Rhyming decision,<br><br>Semantic categorization.	Perceptual decision task- decide if pairs of Greek letter strings were visually identical or different, in a go/nogo task (only respond if strings are visually identical).	Reported stronger and less variable laterality indices for a semantic categorisation versus a rhyming decision task. Frontal dominance indices suggested that this might be explained by a stronger frontal activation in the SC versus the RD task i.e. frontal asymmetries may be stronger for the SC versus the RD task.
Seghier, Kherif, Josse, Price	2011	82	Both right, left and ambidextrous (5)	Semantic word matching task.	Perceptual matching task- match one of two strings of unfamiliar greek letters to target.<br><br>Perceptual matching on photos of unfamiliar non-objects.	The use of a global laterality index is inappropriate given regional heterogeneity in lateralisation, particularly in light of dissociations in laterality across regions, found here between the angular gyrus and ventral precentral gyrus. Indivudal variability in laterality was driven by differences in right rather than left hemisphere activation.
Sepeta, Berl, Wilke, You, Mehta, Xu, et al.	2016	57	Unknown (6)	Auditory description decision task.	Reverse speech with tone identification- press a button when tone follows reverse speech.	Demonstrated moderate laterality within the MTL for a sentence comprehension task, with most subjects showing left lateralisation. MTL laterality was predicted by laterality within Broca's and Wernicke's areas for the same task.
Somers, Neggers, Diederen, Boks, Kahn, Sommer.	2011	22	Both right and left handers (4)	Word generation.	Rest (blank screen, imagine non-verbal items e.g. a starry sky).	Demonstrated moderate laterality for the word generation task in a mixed handedness sample using a single language ROI composed of both frontal and temporal areas.
	2003	12	Only Right handers (1)		Rest/fixation.	

Article authors (article_authors)	Publication year (year_of_ publication)	Sample size (sample_ size)	Sample_Handedness (sample_handedness)	List specific language tasks used (specific_language_ tasks)	Specific baseline tasks used (baseline_tasks)	Key conclusions (study_conclusions)
Sommer, Ramsey, Mandl, Kahn.				Verb generation,<br>Reverse-read semantic decision.		Demonstration of strong left lateralisation obtained for an expressive-receptive combined task analysis in a sample of female right handers.
Stippich, Mohammed, Kress, Hahnel, Gunther, Konrad, et al.	2003	14	Only Right handers (1)	Sematic fluency,<br>Sentence generation.	Fixation.	Demonstration of strong laterality obtained for both a semantic fluency and a sentence generation task across both frontal and temporoparietal ROIs in a sample of right handers.
Suarez, Whalen, O'Shea, Golby	2007	13	Only Right handers (1)	Antonym generation	Fixation.	Laterality depended on method of LI calculation and region. The effect of stimulus modality was region dependent (but generally had no effect). Hemispheric and non-language ROI LIs were highly variable and not significantly lateralised. Significant lateralisation found in IFG and supramarginal gyrus but not in temporoparietal gyrus. SMG LI was less variable and significantly higher than IFG LI for auditory but not visual stimulus presentation.
Sveller, Briellmann, Saling, Lillywhite, Abbott, Masterton et al.	2006	70	Both right and left handers (4)	Verb generation.	Fixation.	Moderately strong laterality found for verb generation task in a large sample with mixed handedness using a combined language ROI. No significant relationship between handedness quotient and LI was found; however a combination of left handedness with typical lateralisation was rare (3/70 subjects).
Szaflarski, Holland, Jacola, Lindsell, Privitera, Szaflarsk.	2008	49	Unknown (6)	Verb generation,<br>Semantic decision.	VG- Bilateral finger tapping in response to tones.<br>SD- Tone decision task, decide if tone sequence contained two tones of a particular frequency.	LIs for a semantic decision task were found to be more left lateralised than those of a verb generation task. May be attributable to the use of an active tone decision baseline for SD (finger tapping used for VG). However, LIs were highly correlated between tasks, across both frontal and temporal ROIs. Frontal LIs were higher than temporal LIs.
Tailby, Weintrob, Saling, Fitzgerald, Jackson	2014	42	Unknown (6)	Word generation,<br>Verb generation.	Rest/fixation	Strong and comparable laterality found for verb generation and word generation tasks; however precise ROI used not known.
Thivard, Hombrouck, du Montcel, Delmaire, Cohen, Samson	2005	17	Unknown (6)	Semantic fluency,<br>Sentence repetition,<br>Story listening.	SF and SR- rest.<br>SL- listen to the same story played backwards.	LI depended on task and ROI. Strongest lateralisation found for story listening in the frontal lobes; but reported that the frontal lobes were inconsistently activated for story listening. The next strongest laterality was found for the fluency task in both frontal and temporal lobes, then story listening in temporal lobes. Sentence repetition was poorly lateralised across both ROIs.
Tie, Suarez, Whalen, Radmanesh, Norton, Golby	2009	6	Only Right handers (1)	Antonym generation.	Rest/fixation.	High degree of discordance between the statistical maps generated by event-related and block designs. Generally, the event-related paradigm yielded more activation within language areas. Difficult to draw conclusions about which gave strongest laterality due to high level of individual variability. Noted that the optimal threshold required by each design may be different.
Tzourio-Mazoyer, Marie, Zago, Jobard, Perchey, Leroux.	2015	281	Both right and left handers (4)	Speech listening.	After listening to speech, subjects had to detect when the central fixation cross changed to a square using a button press response.	Significant right lateralisation found for group mean LI using a speech listening task, global ROI and an event-related design. Explained by high level of individual variability in LI (range: -70 to 80). Lateralisation depended on activity within phonological areas that had strong and opposite asymmetries
Van der Haegen, Cai, Brysbaert.	2012	57	Only Left handers (2)	Word generation,<br>Lexical decision.	For WG: Silent repetition of visually presented non-word (baba)	Demonstration of colateralization between IFG (during word generation) and vOT (during lexical decision) in the majority of subjects in a left handed sample. However, reported a small number of cases (3/57) with crossed dominance.
Van der Haegen, Cai, Seurinck, Brysbaert	2011	50	Only Left handers (2)	Word generation.	Silent non-word repetition (baba).	Strongest laterality found for a word generation task within IFG ROI (both pars opercularis and pars triangularis). Weakest laterality found within the insula. Equal numbers of typical (left) and atypical (right and bilateral) lateralised subjects in this left handed sample (50:50 ratio).
van Oers, Vink, van Zandvoort, van der Worp, de Haan, Kappelle.	2010	13	Only Right handers (1)	Verb generation,<br>Semantic decision,<br>Picture-word matching.	VG- fixation.<br>SD and PWM- press the left or right button when an arrow pointing left or right was presented.	Similar moderately strong laterality found across all tasks and ROIs. Strongest laterality found for picture-word matching in temporal ROI; weakest found for semantic decision in temporal ROI. Greatest difference between posterior and frontal LIs found for verb generation task (0.41 versus 0.47).
van Rijn, Aleman, Swaab, Vink, Sommer, Kahn.	2008	14	Only Right handers (1)	Verb generation,<br>Antonym generation,<br>Semantic decision.	For VG and AG- passive dot pattern viewing.<br>For SD- dot cued button presses.	Demonstration of strong laterality found across anterior and posterior language areas for a CTA consisting of verb and antonym generation and semantic decision. Strongest and least variable laterality found in angular gyrus.
van Veelen, Vink, Ramsey, Sommer, van Buuren, Hoogendam.	2011	43	Only Right handers (1)	Verb generation,<br>Antonym generation,<br>Semantic decision.	WG and AG- passive viewing of squares on screen.<br>SD- perceptual decision (decide if asterisks presented on a screen corresponded to a cue pattern).	Found weak laterality across both a frontal and temporal ROI for a CTA consisting of verb generation, antonym generation and semantic decision in a sample of right handers, using standard LI equation with a variable threshold.
Vassal, Schneider, Boutet, Jean, Sontheimer, Lemaire.	2016	20	Only Right handers (1)	Sentence comprehension decision task.	Letter decision task- decide if pairs of consonant strings were identical or if they differed by just one consonant.	Found strong lateralisation for a sentence comprehension task involving both semantic and syntactic processing using a global LI.
Vikingstad, George, Johnson, Cao.	2000	23	Only Right handers (1)	Verb generation,<br>Picture naming.	VG- passive viewing of five forward slashes.<br>PN- passive viewing of nonsense line drawings.	Moderately strong lateralisation found for verb generation and picture naming tasks. Strongest lateralisation found for verb generation in frontal ROI. Biggest difference between frontal and posterior LIs found for VG. Reported two cases of 'dissociated dominance' involving left lateralisation in one task but bilateral activity in the other (using temporal ROI for naming but frontal ROI for VG).
Vernooji, Smits, Wielopolski, Houston, Krestin, van der Lugt.	2007	20	Both right, left and ambidextrous (5)	Verb generation.	Passive tone listening.	Strength of laterality for verb generation task depended on ROI and handedness; stronger laterality seen for frontal ROIs and for right handers (LI = 0.87). Higher variability in LI values seen for left handers compared to right handers.
Vingerhoets, Alderweireldt, Vandemaele, Cai, Van der Haegen, Brysbaert et al.	2013	20	Both right, left and ambidextrous (5)	Word generation.	Silent repetition of the non-word 'baba'.	Strongest laterality found for a word generation task within a dorsal premotor cortex region, lowest found within posterior parietal cortex. Found some cases in which subjects showed discrepant/incongruous dominance in one ROI as compared to the other four. Overall, laterality across the 5 ROIs (frontal and parietal) was significantly and positively correlated.
Wilke, Lidzba.	2007	12	Only Right handers (1)	Word chain task.	Rest.	Demonstrated no differences between voxel count and voxel value LIs in terms of LI strength or robustness against threshold/statistical outliers. Clustering and variance weighting greatly improved robustness of LI against these. Global LIs were unstable; regional LIs were argued to be more sensitive. Adaptive thresholding may be beneficial.
Tzourio-Mazoyer, Joliot, Marie, Mazoyer.	2016	297	Both right and left handers (4)	Sentence generation	Covert recitation of the months of the year, when presented with a scrambled line drawing (randomly interleaved with non-scrambled drawings for sentence generation).	Found that asymmetries at a regional level were consistent with those at a global level in terms of language dominance. Typicals and strongly atypicals showed mirroring patterns of regional asymmetries; across 50 ROIs, LI was negatively correlated between groups. Differences in asymmetry between the groups were found in both language and non-language ROIs. Demonstration of strong frontal LI for sentence generation task.
Zaca, Jarso, Pillai.	2013	12	Only Right handers (1)	Word generation,<br>Sentence completion,	WG- Passive viewing of nonsense drawings.<br>SC- scan through a series of scrambled letters that resemble words in a sentence.	Word generation was the most strongly lateralising task in the frontal ROIs. An auditory antonym pair decision task was particularly poorly lateralising in the STG and MTG.

**Article authors** (article_authors)	**Publication year** (year_of_ publication)	**Sample size** (sample_ size)	**Sample_Handedness** (sample_handedness)	**List specific language tasks used** (specific_language_ tasks)	**Specific baseline tasks used** (baseline_tasks)	**Key conclusions** (study_conclusions)
				Visual antonym pair,<br><br>Auditory antonym pair,<br><br>Noun-verb association.	vAP- view drawing and decide if simultaneously presented cross is presented in the top or bottom right hand corners of the screen.<br><br>aAP- decide if pair of tones are identical.<br><br>NV- same as vAP.	
