# Supplementary material for: Measuring language lateralisation with different language tasks: a systematic review
Source: PeerJ. 2017 Oct 24;5:e3929. doi: 10.7717/peerj.3929 (PMC5659218; doi:10.7717/peerj.3929)
Supplement: Supplemental Information 7 — A statement of purpose outlining the rationale for the systematic review and its contribution in light of previously published reports. [file peerj-05-3929-s007.docx]

**Appendix 5: Statement of purpose for the systematic review ‘Measuring language lateralisation with different language tasks: a systematic review’**

A key consideration in measuring laterality for language with fMRI is the choice of paradigms for engaging language processing. A wide range of language tasks have been used in imaging laterality research that aim to recruit different language functions, such as speech production, speech comprehension, syntax, semantics and phonology. Comparison of the laterality measured with different language paradigms is of theoretical interest, given suggestions that different language functions may vary in their extent of lateralisation (Rasmussen & Milner, 1975; Hickok & Poeppel, 2007; Price, 2012; Peele, 2012; Bishop, 2013; Poeppel, 2014; Tailby, Abbott & Jackson, 2017). This is in contrast to the view that lateralisation is single dimensional, developing across the entire language network in a unitary manner.

Determining between these different theoretical standpoints is difficult in current laterality research however, due to failures to properly control and systematically vary task design. That is, when comparing between different language paradigms, a lack of control over non-linguistic aspects of task design presents ambiguity when trying to interpret differences in laterality. Such variability could either reflect true differences in the underlying hemispheric organisation of different parts of the language network, or could simply be an artefact of more trivial methodological differences between paradigms. Alternatively, any differences could further reflect a failure to optimise parameters such as the region of interest or type of baseline employed on a task-by-task basis, resulting in less optimal laterality measurement for certain tasks than others.

To our knowledge, there has been no systematic review published of the language paradigms being used in laterality research in terms of their strength and reliability of laterality, and how these may be affected by factors such as region of interest and baseline task. A short review of the general factors that need to be controlled for when calculating an LI was provided by Seghier (2008). This raised task selection as a factor requiring careful consideration, however did not provide any depth of synthesis of evidence on different language paradigms to provide specific recommendations that can be used to guide task selection and development. A recent systematic review by Bradshaw, Bishop and Woodhead (2017) reviewed evidence on the use of different protocols for quantifying laterality from fMRI data, addressing more general methodological issues such as thresholding, region of interest and methods of dominance classification. The current review is intended to complement this previous review, with a focus instead on task design for language laterality measurement.

The aim of this systematic review is to assess evidence on the use of different language paradigms that are widely employed for laterality measurement. We provide a synthesis of evidence on the strength and reliability of laterality measured using each language task, and how this can be affected by linguistic and non-linguistic aspects of the task itself and the laterality protocol. Overall, the purpose of this systematic review is to inform researchers as to how best to measure laterality for different aspects of language functioning in terms of task design, and to encourage the development of closely matched tasks, designed so as to engage distinct aspects of language whilst controlling for non-linguistic factors. This is needed in the field to allow systematic study of differences in laterality across the language network, in order to test recent predictions of multi-dimensionality in language laterality.

**References**

Bishop, D. V. M. (2013). Cerebral asymmetry and language development: Cause, correlate, or consequence? *Science*, 340(6138), 1230531. doi:10.1126/science.1230531

Bradshaw A. R., Bishop D. V. M., Woodhead Z. V. J. (2017) Methodological considerations in assessment of language lateralisation with fMRI: a systematic review. *PeerJ, 5:e3557*. doi: 10.7717/peerj.3557

Hickok, G., & Poeppel, D. (2007). Opinion - the cortical organization of speech processing. *Nature Reviews Neuroscience*, 8(5), 393-402. doi:10.1038/nrn2113

Peelle, J. E. (2012). The hemispheric lateralization of speech processing depends on what "speech" is: A hierarchical perspective*. Frontiers in Human Neuroscience, 6,* 309. doi:10.3389/fnhum.2012.00309

Poeppel, D. (2014). The neuroanatomic and neurophysiological infrastructure for speech and language. *Current Opinion in Neurobiology, 28*, 142-149. doi:10.1016/j.conb.2014.07.005

Price, C. J. (2012). A review and synthesis of the first 20 years of PET and fMRI studies of heard speech, spoken language and reading. *Neuroimage*, 62(2), 816-847. doi:10.1016/j.neuroimage.2012.04.062

Rasmussen, T., & Milner, B. (1975). Clinical and surgical studies of the cerebral speech areas in man. In K. J. Zulch, O. Creutzfeldt & G. C. Galbraith (Eds.), *Cerebral localization* (pp. 238). Berlin: Springer.

Seghier, M. L. (2008). Laterality index in functional MRI: Methodological issues. *Magnetic Resonance Imaging*, 26(5), 594-601. doi:10.1016/j.mri.2007.10.010

Tailby, C., Abbott, D. F., & Jackson, G. D. (2017). The diminishing dominance of the dominant hemisphere: Language fMRI in focal epilepsy. *NeuroImage*: Clinical, 14, 141-150. doi.org/10.1016/j.nicl.2017.01.011
